# Neutral network sizes of biological RNA molecules can be computed and are not atypically small

**DOI:** 10.1186/1471-2105-9-464

**Published:** 2008-10-30

**Authors:** Thomas Jörg, Olivier C Martin, Andreas Wagner

**Affiliations:** 1 Inria Saclay – Ile-de-France, INRIA, Parc Orsay Université 4, rue Jacques Monod 91893 ORSAY Cedex, France; 2 Laboratoire de Physique Théorique et Modèles Statistiques, Université Paris-Sud, 91405 Orsay Cedex, France; 3 UMR0320/UMR8120 Génétique Végétale, Université Paris-Sud, F-91190 Gif-sur-Yvette, France; 4 Department of Biochemistry, University of Zurich, Winterthurerstrasse 190, CH-8057 Zurich, Switzerland; 5 The Santa Fe Institute, 1399 Hyde Park Road, Santa Fe, NM 87501, USA; 6 Swiss Institute of Bioinformatics, Quartier Sorge, Batiment Genopode, 1015 Lausanne, Switzerland; 7 University of New Mexico, Department of Biology, 167 Castetter Hall, Albuquerque, MSC03 2020, USA

## Abstract

**Background:**

Neutral networks or sets consist of all genotypes with a given phenotype. The size and structure of these sets has a strong influence on a biological system's robustness to mutations, and on its evolvability, the ability to produce phenotypic variation; in the few studied cases of molecular phenotypes, the larger this set, the greater both robustness and evolvability of phenotypes. Unfortunately, any one neutral set contains generally only a tiny fraction of genotype space. Thus, current methods cannot measure neutral set sizes accurately, except in the smallest genotype spaces.

**Results:**

Here we introduce a generalized Monte Carlo approach that can measure neutral set sizes in larger spaces. We apply our method to the genotype-to-phenotype mapping of RNA molecules, and show that it can reliably measure neutral set sizes for molecules up to 100 bases. We also study neutral set sizes of RNA structures in a publicly available database of functional, noncoding RNAs up to a length of 50 bases. We find that these neutral sets are larger than the neutral sets in 99.99% of random phenotypes. Software to estimate neutral network sizes is available at .

**Conclusion:**

The biological RNA structures we examined are more abundant than random structures. This indicates that their robustness and their ability to produce new phenotypic variants may also be high.

## Background

Every cell is packed with solutions to the problems its ancestors faced. These solutions are embodied in biological macromolecules – RNA and proteins – which produce energy from nutrients, neutralize external stressors, coordinate cell division, defend cells against invaders, and so on. Most of us think of these solutions as extremely rarefied: They would be difficult to find in the space of possible nucleotide or amino acid sequences, because they occupy exceptionally small regions in this space. Their discovery by living things was hard-won, through innumerable generations of mutation and natural selection. Despite this common wisdom motivated by stringent functional constraints on biological molecules, we have little rigorous, quantitative understanding of how abundant or rare the molecular structures of biological molecules are. The fundamental reason is that our ability to characterize genotypes (sequences) still vastly exceeds our ability to characterize phenotypes (molecular structures and functions). While it is simple to determine the nucleotide sequence of a gene and even of entire genomes, the prediction of the structure of individual proteins or RNA molecules, let alone of their integrated behavior, is a major challenge.

If they are extremely rare, functional phenotypes may be very difficult to find in a blind evolutionary search. However, the significance of phenotype rarity does not end with this observation. The descendants of biological macromolecules may give rise to molecules with new phenotypes and functions – evolutionary innovations. The ease with which they do is also called their evolvability [1]. Some molecules have been extremely prolific in this regard – highly evolvable – whereas others have been less so. The rarity of a molecule may affect its propensity to evolve new structures and functions. To see why, it is useful to consider that such molecules are usually part of large networks of genotypes [2-6]. Most known structures of protein and RNA molecules are adopted not by one sequence, but by large sets of sequences. Many or all of these sequences can be connected in sequence space through series of nucleotide or amino acid changes that traverse a large fraction of this space, yet leave the structure and function of the molecule unchanged. Such sets of sequences are often referred to as neutral sets or neutral networks [2]. Specifically, a neutral set is a set of sequences with the same phenotype. A neutral set is called a neutral network if all sequences in it can be connected through series of single mutations that do not leave this set. This distinction maintains the generality of our framework. However, for the RNA phenotypes we study neutral sets are almost always connected, so the two terms can be used interchangeably. The size of a neutral set is a measure of a phenotype's rarity in sequence space. The greater this size, the easier it should be to find the phenotype in an evolutionary search. We will refer to phenotypes with large neutral sets as abundant or frequent phenotypes.

Evolutionary innovations arise when mutations that explore variants of a functional phenotype strike a molecule with a new and useful function. A large neutral network can be of advantage in this process, because the immediate neighborhood of a large neutral network in sequence space contains many more phenotypic variants than that of a smaller neutral network. Through neutral evolution on large neutral networks, molecules can thus get access to many molecular variants. This is why high abundance of a phenotype can be argued to be beneficial for evolutionary innovation [7]. Recent evolutionary work on protein structures shows that abundant protein phenotypes have indeed evolved greater functional diversity [8]. Other factors, such as neutral network topology may also play a role in evolutionary innovation [9-11].

These observations motivate the need for approaches to estimate the abundance of phenotypes in sequence space. We here show how to solve this problem for a computationally accessible molecular phenotype, the secondary structure of RNA molecules. RNA secondary structure is required for the biological function of many RNA molecules [12-14]. It is thus an important phenotype in its own right. Because algorithms to predict RNA secondary structure from an RNA sequence are available [15-17] secondary structure is an important computational model to understand the relationship between RNA genotypes and phenotypes [2,4,10,18]. The computational challenge to estimate whether an RNA phenotype is frequent or rare, i.e., whether it is adopted by many or few sequences, is formidable. For example, even for sequences of length *L *= 50 one has to estimate numbers smaller than 10^-15 ^(expressed as a fraction of the size 4^L ^of sequence space). Below, we discuss the details of the method we developed, which is based on a nested sampling of genotypes. We then apply this method to multiple biological and random RNA sequences. The results demonstrate that biological RNA structures have a large number of sequences that fold into them, much larger than for random phenotypes. This number of sequences may also be moderately larger than for structures produced from random genotypes.

## Methods

### Software for structure prediction and inverse folding

For our analyses, we used the Vienna RNA package (; [15]), including the routines fold, which determines the minimum free energy (mfe) structure of a sequence, and inverse_fold, which creates sequences folding into a given minimum free energy structure, using a guided random walk through sequence space that begins with a randomly chosen sequence. We also used the utility bp_distance which calculates the base-pair distance of two arbitrary structures.

### Sampling neutral sets

In the literature, heuristic sampling of neutral sets has been performed by using the inverse_fold routine implemented in the Vienna RNA package (; [15]). To test this approach, we studied its statistical bias by comparing its results to results obtained by random sampling of compatible sequences – sequences where only bases capable of pairing occur in the structure's stacks. We found that inverse_fold does not sample the desired set of sequences uniformly: sequences that are on the "boundary" of this set are sampled more frequently. We thus refrain from using this approach when uniform random sampling is necessary. For our study, the routine inverse_fold is used only in the initialization of the Monte Carlo approach which quickly loses the memory of this initial choice.

### Error estimates

To estimate a neutral set size for any given structure *S*, we first carry out a very long run of the Nested Monte Carlo procedure (at least 10^5 ^cycles of mutation and exchange at each *d*) to estimate the size of the neutral set. We measure the quantities *O*_*i *_= *χ*_*d*_(*G*)(*i *= 1, ... 50) that are the averages in each of the fractional intervals [(*i *- 1) × 0.02, *i *× 0.02] of the total run. That is, each *O*_*i *_is computed from a fraction of 2% of the total run. This gives 50 estimates of neutral set size, where the global average is the actual estimated size (e.g., Table 1). We then use the 50 values of *O*_*i *_to obtain the error in this estimate. As in all Markov chain Monte Carlo methods, the 50 values *O*_*i *_are not independent. To address this problem, we apply the jackknife method [19] which is a general way to compute errors even for correlated and non-normally distributed data. In this method, if one has *k *samples *O*_*i*_, one first computes *k *averages *m*_*i *_of these samples, omitting for each average the single value *O*_*i*_. The resulting set of values (*m*_1_, ..., *m*_*k*_) has some standard deviation *σ*. The jackknife error estimate is given by $\sqrt{k-1}\sigma$. It is that value we report as the error bar on the neutral set size estimates.

In a similar vein, we obtained error estimates for *P*-values as follows, again using the jackknife method. A structure's *P*-value is determined from expression (10) for a sample of *M *phenotypes. For each *i *= 1, ..., *M*, we remove the *i*-th structure from this sample and recompute the P-value according to (10) with this altered sample. If *σ *is the standard deviation of these *M *estimates, then the Jackknife procedure specifies $\sqrt{M-1}\sigma$ as the error of these estimates; this is the error we quote in Table 1.

## Results

To determine neutral network sizes, one can in principle enumerate all sequences and the structures they fold into, or one can sample by ''brute force'' many sequences from sequence space, and estimate the fraction of sequences with a structure of interest. If one focuses only on sequences compatible with a given structure – sequences where only bases capable of pairing occur in the structure's stacks – then these approaches are practical for single structures up to *L *≈ 40. However, for our work we need to do this kind of calculation for thousands of structures, and for neutral set sizes that may exceed 10^20^. We thus need a more sophisticated approach. In what follows we describe a method that leads to reliable estimates for much larger *L*. In addition, this method achieves uniform sampling regardless of whether sequences adopting a given structure fall into one neutral network, or into multiple, disjoint neutral networks. In a second part, we explain how this approach can be used to quantify whether a structure's neutral set is atypically large or small.

### Part 1: A Nested Monte Carlo approach to estimate the size of a neutral set

We are given a discrete space of *4*^*L *^genotypes (RNA sequences), where *L *is sequence length. We would like to determine the number of genotypes in this space that have a given "target" phenotype (structure) S*. To this end, we have developed a Monte Carlo sampling approach. It builds on the Metropolis algorithm [20] that can sample connected spaces according to any predefined probability measure. However, the sampling of a set does not yield an estimate of its size. We overcome this shortcoming by considering nested sampling. Our approach only assumes that there exists a distance metric *d*(*S*, S*) among all phenotypes, or at least a measure of distance between any phenotype *S *and the target S*. (We here used the base-pair or bond distance calculated in the Vienna RNA package [15], and note that the choice of distance does not affect the framework of our estimation method.) In practice, *d *will be an integer, ranging from 0 to some integer *d*_*max*_; *d *= 0 if and only if *S *= S*. We will call *V*(*d*) the number of genotypes whose phenotype *S *satisfies *d*(*S*, S*) ≤ *d*, and we will refer to the set of these genotypes also as *V*(*d*). Our quantity of interest is *V*(0), the size of S*'s neutral set, be it connected or not. To compute this quantity, we use the identity

(1) $$V(0)=\frac{V(0)}{V(1)}\frac{V(1)}{V(2)}\ldots \frac{V(d_{\max}-1)}{V(d_{\max})}V(d_{\max})$$

Because *V*(*d*_*max*_) = 4^*L *^is known, *V*(0) can be estimated from the estimates of all the ratios *V*(*d*)/*V*(*d*+1). These ratios can be estimated using the Metropolis algorithm by sampling uniformly the space *V*(*d*+1), and measuring the average of the indicator (characteristic) function *χ*_*d *_of *V*(*d*)

(2) $$\begin{array}{l} \chi_{d}(G)=1\;\text{if }G\in V(d)\\ \chi_{d}(G)=0\;\text{if }G\notin V(d) \end{array}$$

Note that by construction the sets *V*(*d*) are nested, either like Russian dolls, or in more complicated ways, since each set need not be connected. The innermost set, *V*(0), is the ultimate set of interest and its size is the desired neutral set size; all the other sets are just of use to connect *V*(0) to the known quantity *V(d*_*max*_) = 4^*L*^.

To estimate the ratio *V*(*d*)/*V*(*d*+1) we sample *V*(*d*+1) uniformly using the Metropolis algorithm. Specifically, we begin with an arbitrary genotype *G*_1 _in *V*(*d*+1), and produce a (Markov) chain, *G*_1_, *G*_2 _... *G*_*k *_..., of genotypes. To obtain *G*_*k*+1 _from *G*_*k*_, a random nucleotide in *G*_*k *_is changed, producing a mutated genotype *G*'; if *G' *∈ *V*(*d *+ 1), then *G*_*k*+1 _= *G*', otherwise *G*_*k*+1 _= *G*_*k*_. At sufficiently large *k*, the distribution of *G*_*k *_is uniform in *V*(*d*+1), allowing for unbiased statistical estimates of *χ*_*d*_(*G*). To be precise, at this stage the sampling is uniform but restricted to the connected component of *V*(*d*+1) that contains *G*_1_.

As described so far, nested sampling estimates the ratios *V*(*d*)/*V*(*d*+1) by performing independent Monte Carlo simulations for each *d*, but the algorithm is sound only if each set *V*(*d*) is connected. To guarantee soundness even when this is not the case, we estimate all the ratios in (1) simultaneously, introducing genotype ''swaps'' similar to those used in the Exchange Monte Carlo approach [21,22]. Specifically, we first initialize the Monte Carlo procedure by establishing as many sequences in *V*(0) as there are ratios to estimate in (1). It is simplest to initialize all these sequences to the same element of the neutral set which we assume to be non empty. Whether this initial sequence is from an unbiased (i.e., uniform) distribution does not matter. We thus use inverse_fold [15] to establish such a sequence. Each of these sequences will then start a random walk that will be used to estimate one of the ratios of (1). At each round of the Nested Monte Carlo, there are now two steps. The first is a mutation step, in which each random ''walker'' is mutated as described above according to the Metropolis rule; it is thus confined to the set *V*(*d*+1) used to estimate the ratio *V*(*d*)/*V*(*d*+1) (*d *is different for every random walker). The second step consists of a swap of two sequences: genotype 1 in *V*(*d*) is exchanged with genotype 2 in *V*(*d*+1), if and only if genotype 1 also lies in *V*(*d*+1), and if genotype 2 also lies in *V*(*d*). That a genotype lies in two sets is possible, because the sets are nested. Just as in Exchange Monte Carlo, one can prove that the detailed balance condition upon with the success of the Metropolis algorithm rests [20] is still satisfied with this procedure; thus the desired fractions can still be computed in the same way as for the simple sampling previously described.

While it may seem that this generalized Monte Carlo method is simply a parallel version of our initial sampling, the introduction of swaps has two important benefits. First, as in all Markov chains, the successive sequences of genotypes generated in the Metropolis algorithm are correlated. This correlation leads to statistical errors and thus is undesirable. The random swaps reduce this correlation and thus lead to greater computational power. Second, ergodicity – uniform sampling regardless of whether the sets *V*(*d*) are connected or not – is guaranteed by the modified Monte Carlo algorithm. The reason is as follows. The detailed balance condition for each walker ensures that all genotypes which can be reached are necessarily sampled with equal probability. Now in the largest volume *V(d*_*max*_) (the entire sequence space), the random walk is ergodic, simply because the entire sequence space *V*(*d*_*max*_) is connected. Through swaps, walkers can reach any genotype in *V*(*d*_*max*_-1), so that the random walk in *V*(*d*_*max*_-1) is also ergodic. By recurrence, one can see that the random walk in *V*(*d*) is ergodic for all *d*.

We note that the sampling scheme from (1) can also be generalized to other nested sets of volumes that do not use successive values of *d *as in (1). Greater efficiency could be obtained by adapting the choice of *d*-values: Having too many fractions to estimate in (1) leads to excessive computational cost, while too few fractions lead to poor sampling and large sampling variance. In our application to RNA molecules below, we found that the simplest procedure, of using all *d *up to *d*_*max *_was adequate. Note also that our approach will work not only for RNA genotypes, but for any genotype space (discrete or continuous) as long as a distance metric between phenotypes exists. Our software to estimate neutral network sizes is available at .

### Part 2: Evaluating the abundance of secondary structures

We now have described how to estimate the neutral set size of an individual structure.

One of our goals is to find out whether biological structures have neutral sets that are atypical in size. Since evolvability arguments suggest that these sizes might be large, we shall ask whether biological neutral network sizes are much larger than those of typical structures. Specifically, we wish to test the null hypothesis *H*_0 _that a given secondary structure of a biological RNA molecule has an associated neutral set whose size could have been drawn at random from the distribution of all neutral set sizes, i.e., from randomly chosen *phenotypes*. (Further below, we shall also briefly consider phenotypes generated from random *genotypes*.) This task requires us to estimate neutral set sizes for *many *different structures. However, already for moderate length *L*, there is an astronomical number of structures, and we thus cannot enumerate them exhaustively. We here demonstrate the theoretical foundation of an enhanced sampling method that allows us to estimate the comparative abundance of a phenotype.

Neutral set sizes *N*_*S *_follow some distribution *P(N*_*S *_= *x*), defined as the probability that *N*_*S *_equals some integer *x *(*x*_*min *_≤ *x *≤ *x*_*max*_). Although this distribution is discrete, there are so many different structures that a continuous notation with a corresponding probability density *ρ*(*x*) is appropriate. Note that $\int_{0}^{\infty }\rho (x)dx=1$. We would reject *H*_0 _if, for a specific phenotype S* and its neutral set size *N*_S*_,

(3) $$P(S*)=\int_{N_{S*}}^{\infty }\rho (x)dx<0.05$$

i.e., we integrate over the right tail of the distribution, thus performing a one-tailed test. If (3) holds for a neutral set, we call the set atypically large at a confidence level of 0.05, but this threshold can of course be reduced if a more conservative test is needed.

We next demonstrate an intimate link between the *P*-value and the rank histogram of neutral set sizes, which will lead us to a sampling scheme to estimate small *P*-values.

**Figure 1:**
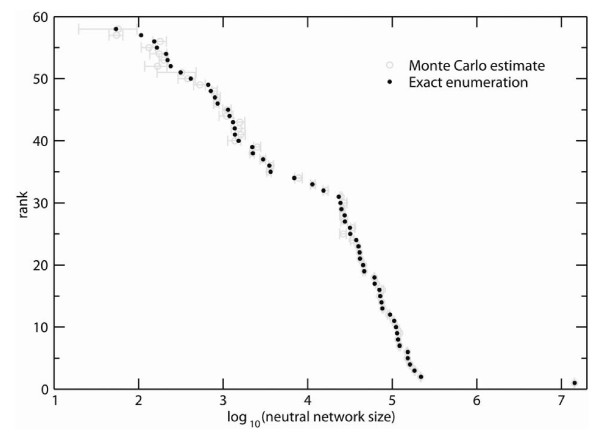
**Validation of algorithm**. For all 58 structures adopted by sequences of length 12, the horizontal axis shows neutral network sizes, the vertical axis shows the rank of each structure, as determined by neutral network size. This rank was determined in two different ways, by exact enumeration (black, solid circles), and by the Nested Monte Carlo approach (grey circles, error bars) described in the text.

For very short sequences, one can calculate *P*-values by exhaustive enumeration of sequences and structures. Consider, for example Figure 1, which shows all 58 RNA secondary structures for *L *= 12 for which there exists at least one sequence folding into the structure. (We never consider structures for which the neutral set is empty, i.e. *N*_*S *_= 0.) In this case with *L *= 12, each neutral set (network) size is unique. In the figure, the structures are rank-ordered with the largest neutral network size (lowest rank of 1) to the right. For any given structure, we can immediately evaluate whether (3) holds by verifying whether it is among the 5% of phenotypes with lowest rank. More precisely, if *N *is the total number of structures, and *R *is the rank of a given structure S*, then the associated *P*-value can also be thought of as a "relative rank" *P*(S*) = *r*: = *R*/*N*. Ties, where two or more structures have the same neutral set size, can be resolved by assigning these structures successive ranks. Note that the most abundant, lowest ranked structure in Figure 1 corresponds to the unfolded "structure". Because that structure is of no interest for our work, we shall not include it in our figures or data sets hereafter.

For large *L*, such rank histograms cannot be computed, because the number of structures scales exponentially with *L*, so it is not generally possible to identify all structures. Our sampling approach avoids this problem, thereby allowing the estimation of *P*-values at much larger *L*. The key point is that the (absolute) rank of a structure is not necessary, we only need an estimate of its relative rank, and that can be obtained as follows. First, we generate M random structures, where each structure is obtained with equal probability, compute their neutral set sizes, and then sort these sizes. For the second step, consider a structure S* of neutral set size *N*_S*_. Its (absolute) rank *R *is unknown, but its relative rank *R/N *can be estimated as *R'/M *where *R' *is the number of structures *in the sample of size M *that have neutral sets at least as large as *N*_S*_. The associated estimate of *P*(S*) is then simply *R'*/*M*.

A complication to this sampling approach comes from the requirement of random (uniform) sampling of *phenotypes*. For RNA secondary structures, phenotypes could be sampled by random assignments of allowed base pairings [23], but in other systems, such phenotypic sampling may not be straightforward. In addition, some phenotypes may have empty neutral sets, i.e., *N*_*S *_= 0, in which case the phenotype is not "designable" [15,23-25]. Undesignable phenotypes are of limited biological interest, but certain knowledge that a phenotype is undesignable is hard to come by. To overcome this challenge, and to avoid undesignable phenotypes, one can perform random sampling of *genotypes *instead. However, in this approach, the computation of the *P*-value has to be modified because one does not sample phenotypes uniformly, but only genotypes. In effect, each phenotype *S *is chosen with a probability $N_{S}/\sum_{S}N_{S}$ that is linearly proportional to the size *N*_*S *_of its neutral set. In such a sampling, phenotypes with large neutral sets arise more frequently than those with small neutral sets. In this sense, the sampling of phenotypes is biased (non-uniform). Incidentally, this bias focuses the sampling on structures with large neutral sets, which allows us to estimate small *P*-values with a small statistical error. We next explain how to calculate the *P*-values of equation (3) with this sampling scheme.

Returning to our continuous notation, denote by *μ*(*x*)*dx *the probability of obtaining a neutral set size in the interval [*x*, *x*+*dx*] through this random genotype sampling. The linear dependence of the sampling probability on neutral set size leads to the following expression for *μ*(*x*):

(4) $$\mu (x)=\frac{x\rho (x)}{\int_{0}^{\infty }y\rho (y)dy}$$

The denominator is a normalization constant which ensures that *μ*(*s*) is a proper probability density. Equation (3) can be rewritten as

(5) $$P(S*)=\int_{N_{S*}}^{\infty }\mu (x)\frac{\rho (x)}{\mu (x)}dx$$

It follows from (4) that

(6) $$\frac{\rho (x)}{\mu (x)}=\frac{1}{x}\int_{0}^{\infty }y\rho (y)dy$$

Taking advantage of (6) to modify (5) yields

(7) $$P(S*)=\int_{N_{S*}}^{\infty }\mu (x)\frac{1}{x}\int_{0}^{\infty }y\rho (y)dydx=\left(\int_{N_{s*}}^{\infty }\mu (x)\frac{1}{x}dx\right)\left(\int_{0}^{\infty }y\rho (y)dy\right)$$

We can determine the value of the rightmost integral by setting *N*_S* _to zero, because Equation (3) shows that in this case *P*(S*) = 1. We then obtain

(8) $$\int_{0}^{\infty }y\rho (y)dy=\frac{1}{\int_{0}^{\infty }\mu (x)\frac{1}{x}dx}$$

Finally, substituting (8) into (7) gives

(9) $$P(S*)=\frac{\int_{N_{S*}}^{\infty }\mu (x)\frac{1}{x}dx}{\int_{0}^{\infty }\mu (x)\frac{1}{x}dx}$$

In sum, we can use (9) to estimate *P*(S*) by sampling genotypes at random (which is equivalent to sampling phenotypes with a probability density given by equation (4)). In practical terms, in order to estimate the *P*-value of any (biological) structure S* of interest, we first determine the neutral set sizes (*N*_*S*1_, ..., *N*_*SM*_) for *M *structures obtained from a sample of *M *random sequences, using the Nested Monte Carlo approach. We then estimate the structure's neutral set size *N*_S*_. Finally, we estimate the *P*-value of S* as

(10) $$P(S*)\approx \frac{\sum_{\left\{i|N_{s_{i}}>N_{s*}\right\}}\frac{1}{N_{S_{i}}}}{\sum_{i=1}\frac{1}{N_{S_{i}}}}$$

Here, summation in the numerator extends over all structures in the sample whose neutral set is greater than that of S*.

Analogous *P*-values can be estimated for related hypotheses. For example, beyond testing for anomalously small neutral set sizes, one can ask whether the neutral network of a particular phenotype is significantly larger than neutral networks associated with the phenotypes of random *genotypes*; to test this hypothesis, no reweighting is necessary and *P*(S*) is simply given by the fraction of random genotypes that have neutral sets larger than S*. Additional File 1 shows a comparison of our procedure with an exact enumeration method that is tractable for very short sequences.

### Algorithm performance

The Nested Monte Carlo approach overcomes the difficulty of measuring the tiny fraction *V*(0)/*V*(*d*_*max*_) by replacing it with the problem of measuring the series of larger fractions *V*(*d*)/*V*(*d*+1). The cost paid is the need to follow *d*_*max *_random walkers rather than just one such walker. For our RNA application, this cost is dominated by the cost of folding sequences. In the Vienna package [15], the time to fold a sequence of *L *bases grows as *L*^3^. This is to be compared with the time to implement a random mutation (*O*(1)) or to implement a swap (*O*(*L*)). It is thus no surprise then that the Nested Monte Carlo procedure consumes nearly all its CPU time within the folding routine. In an individual run, at least 10^5 ^× *L *mutations are carried out. On today's standard desktop workstations (AMD Opteron, 2.4 GHz) it takes approximately 30 minutes to compute the neutral network size to within 2% when *L *= 30, about 145 minutes when *L *= 50, and more than 24 hours when *L *= 100. The longer the run, the more precise the estimate becomes.

We have the choice of sampling the whole space of genotypes, or of imposing any additional constraint on the genotypes, as long as *V*(0) (the neutral set size, *N*_*S*_) is unaffected and the restricted *V*(*d*_*max*_) can be computed. We thus implemented in our software tool the ability to impose the constraint of working only with "compatible" sequences. We here use this ability. Specifically, we force those bases which are paired in *V*(0) to always be "compatible" i.e., the pairs A-C, A-G and C-U are not allowed. This constraint leads to a smaller sampling space in our nested Monte Carlo approach, and thus to a smaller statistical error.

Since the sampling is performed via a Markov chain, the successive genotypes are highly correlated, because they differ by only one mutation. One can observe these correlations very clearly via the distance between a genotype at mutation/swap cycle *t *and the genotype at cycle *t*+*τ*. These correlations are expected to persist on a time scale that is on the order of the number *L *of bases of the sequence. The reason is that each base should be mutated at least once, if the distances are to decorrelate completely; the inset of Additional File 2 validates this expectation. Clearly, a Monte Carlo run must be much longer than this decorrelation time, and even in that situation the statistical error analysis requires some care. For illustration, we display in Additional File 2 the estimator of *V*(0) as a function of the cycle number, using window averages. The signal is clearly noisy and on this time scale the short term memory (correlation) is invisible.

### Application to biological RNA sequences

We next applied the Nested Monte Carlo algorithm to 82 sequences of length 30 ≤ *L *≤ 50 in the functional RNA database fRNAdb [, ref [26]]. The database does not provide curated structures, so we used the secondary structures predicted by the Vienna package [15]. Only computational limitations prevented us from studying a larger data set or a data set of longer sequences. We determined both neutral set sizes and *P*-values for secondary structures, where a structure's *P*-value is, as defined above, the fraction of structures with a larger neutral set. Table 1 shows one representative from each functional category in this data set. It is evident that even for the relatively short sequences considered here, neutral set sizes are enormous. For example, there are more than 10^22 ^sequences forming the predicted structure of a snRNA of *Pyrococcus abyssi *(genbank ID AJ248287). However, even the collection of all these sequences constitutes only a small fraction (1.1 × 10^-9 ^= 1.1 × 10^22^/4^50^) of the vast sequence space. The computation of the *P*-value of this structure gives 2.95 × 10^-5^. This means that fewer than one in 30,000 (1/2.95 × 10^-5 ^= 33,898) structures have a larger network than this structure. Similar *P*-values arise for the other structures in Table 1. We note that the error estimates of both neutral set sizes and *P*-values are generally substantially smaller than the estimates themselves, that is, the relative error is small. In the Supplementary Material (Table S1), we give the neutral network sizes for all 82 sequences examined.

**Figure 2:**
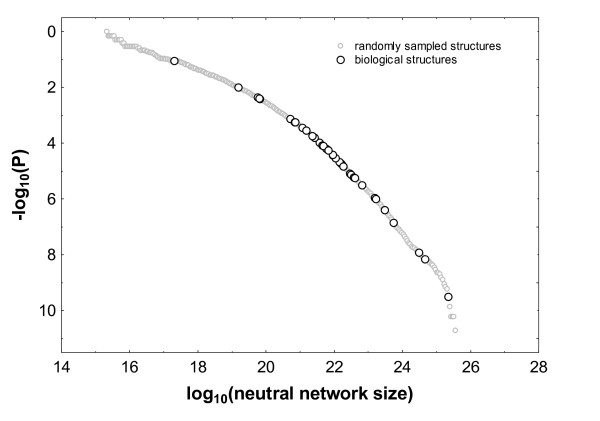
**Biological RNA molecules have atypically large neutral networks**. a) The horizontal axis shows neutral network sizes, the vertical axis shows *P*-values determined for a random sample of 5000 RNA structures of length *L *= 50 (grey circles), as well as all 38 RNA molecules of length *L *= 50 from the functional RNA database (black circles) [26]. b) distribution of *P*-values, and c) distribution of neutral network sizes, for structures in the functional RNA database with length L ≤ 50.

Figure 2a shows a comparison of neutral network sizes and *P*-values for randomly (i.e. uniformly) chosen RNA secondary structures and biological RNA sequences. The data for randomly chosen structures were obtained by sampling 5000 random sequences of length *L *= 50, and determining their neutral network size and *P*-value as explained above. Superimposed are the corresponding data for 38 biological RNA sequences of length *L *= 50. Neutral network size estimation errors have been omitted for clarity, but the median relative error did not exceed 2%. The median neutral network size among the biological structures shown is 9.1 × 10^21^, with a 10^*th *^percentile of 6.4 × 10^19 ^and a 90^*th *^percentile of 5.54 × 10^23^. The median biological structure in this data set comprises a fraction 7.2 × 10^-9 ^of sequence space. The median *P*-value is 3.81 × 10^-5 ^(10^*th *^percentile: 1.4 × 10^-7^; 90^*th *^percentile: 4.03 × 10^-3^). This means that, on average only one in 26,247 (= 1/3.81 × 10^-5^) random structures have a neutral network greater than biological structures in this data set.

An analogous analysis can be performed by comparing the neutral network sizes of biological structures to neutral network sizes of random *genotypes*. Random genotypes adopt phenotypes whose neutral network sizes are larger than that of random phenotypes, because each phenotype is produced with a probability proportional to the size of its neutral network (see also the formulae in Part 2). In this analysis, we find that for random sequences of length *L *= 50, the associated median neutral network size is 3.64 × 10^21 ^while the 90^*th *^percentile is 2.87 × 10^23^. Thus the biological sequences we studied have larger neutral networks than random sequences, but the difference is less dramatic than for random phenotypes, and our sample sizes are too small to make statistical conclusions.

Because of the different sizes of sequence spaces for different *L*, rank histograms like that of Figure 1 cannot be produced for sequences mixing different lengths. However, *P*-values can be compared for such sequences, because their meaning is length-independent. Figure 2b shows a histogram of logarithmically transformed *P*-values for all 82 (Additional File 3) structures examined here. Again, this larger data set also shows that biological structures have atypically large neutral networks when compared to random structures. The median *P*-value for all 82 structures is 5.7 × 10^-5^, with a 10^th ^and 90^th ^percentile of 4.1 × 10^-7 ^and 4.4 × 10^-3^. In sum, fewer than one in 10,000 randomly chosen structures have more associated sequences than the typical biological RNA structure in our data set. Only one out of 82 structures has a *P*-value of greater than 0.05, and only four have a P-value greater than 0.01. Figure 2c shows, for the same 82 structures, a histogram of neutral network sizes, expressed as fractions of sequence space. As in the above examples, the neutral networks of even such highly abundant structures span only a tiny fraction of sequence space. (Median/10^th^/90^th ^percentile:1.4 × 10^-7^/9.2 × 10^-10^/9.6 × 10^-5^). This can be understood from the fact that even the set of sequences compatible with a secondary structure, which contains the neutral network, encompasses only a tiny fraction of sequence space [4,27].

**Figure 3:**
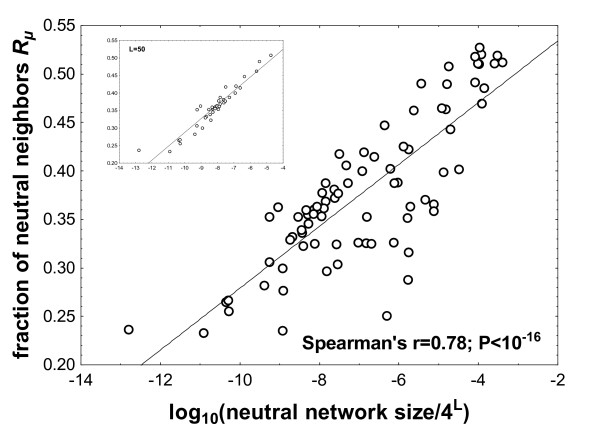
**Mutational robustness correlates with neutral network size**. The horizontal axis shows the logarithm of neutral network sizes divided by *4*^*L *^for the biological RNA sequences examined here. The vertical axis shows *R*_*μ*_, the average mutational robustnes of sequences belonging to a neutral network (see main text for definition of mutational robustness). Each data point for *R*_*μ *_is based on 40000 sequences obtained through the uniform sampling of one neutral set using the Nested Monte Carlo procedure. The inset shows only data for biological sequences with the same length of *L *= 50. The diagonal line was obtained by linear regression

The mutational robustness of a *sequence *is the fraction of its neighbors that are neutral (have the same phenotype as it), or, equivalently, the fraction of mutations that leave a sequence's structure unchanged [28,29]. Similarly, we can define the mutational robustness *R*_*μ *_of a *structure *as the mean mutational robustness of the sequences belonging to its neutral network. Figure 3 shows how *R*_*μ *_depends on neutral network size. For the 82 biological RNA sequences we examined, mutational robustness increases (Spearman's *r *= 0.78) with increasing logarithm of the neutral network size, *N*_*S*_. If we focus on structures of a given length *L*, this association is even stronger (e.g., for *L *= 50 Spearman's r = 0.95; n = 38; P < 10^-17^; Figure 3, inset). The partial correlation coefficient between the two quantities (controlling for length) is r = 0.92 (P < 0.05). We also observe that as neutral network size increases by eight orders of magnitude (note the logarithmic scale on the horizontal axis of Figure 3), mutational robustness increases only modestly, i.e., by a factor of approximately two.

**Figure 4:**
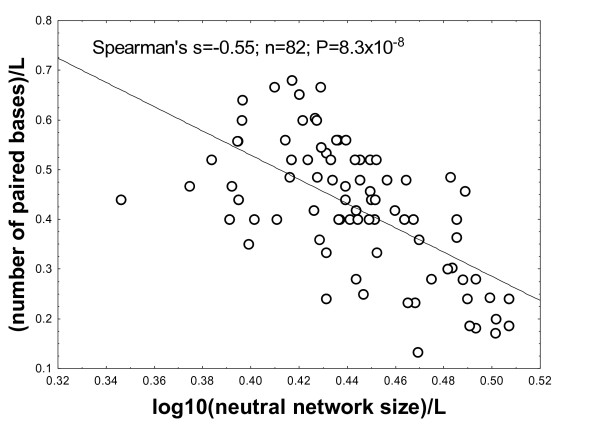
**Other indicators correlate modestly with neutral network size**. The horizontal axes show the logarithm of neutral network sizes for the 82 biological RNA sequences examined here, divided by their length *L*. a) The vertical axis shows the number of paired bases in each of these sequences divided by *L*. b) The vertical axis shows the contiguity statistic [33] described in the main text. The diagonal lines were obtained by linear regression.

Finally, given the computational cost of our Nested Monte Carlo approach, it is reasonable to ask whether there are good indicators of neutral network size that are more easily computed. Possibly the simplest candidate indicator is the number of paired bases in a structure. In line with the simple expectation that each base has a certain probability of being paired in a random structure, one finds empirically that the mean number of paired bases grows linearly with *L*. Similarly, the entropy of a structure, defined thermodynamically as the logarithm of the neutral network size *N*_*S*_, is expected to grow linearly with *L*. As a consequence, to compare indicators of neutral network size across structures of different length *L*, it is useful to compare these quantities to their mean or median values. For that reason, we consider the association between log(*N*_*S*_)/*L *and the fraction of paired bases. We find a significant negative association (Figure 4a; Spearman's *r *= -0.63; P < 10^-9^). The more paired bases a structure has, the smaller is thus its neutral network. However, this association explains less than 40% of the variance of neutral network size (coefficient of determination *r*^2 ^= 0.39). We note that omitting the length-normalization of neutral network size or the number of paired bases leads to even lower associations. Previous work, partly based on artificial random graphs, partly based on genotype-phenotype maps of short sequences, points to reasons why such indicators have limited value [27-32]. It also indicates that the minimum free energy itself may be an indicator of the biological origin of a structure [30].

Recently, an easily computed contiguity statistic of neutral network sizes was proposed [33]. This indicator adds a structure's total bases in stem-loops to the number of paired bases, and divides this sum by the number of stacks. We find that this indicator is positively associated with neutral network size (Figure 4b; Spearman's *r *= 0.36; *n *= 82; P = 1.05 × 10^-3^), an association that decreases if neutral network size is not length-normalized. The association explains a fraction *r*^2 ^= 0.16 of the variance. Our observations above suggest that the biological RNA phenotypes we examined differ very significantly from random phenotypes, which raises the possibility that the previous indicators may work better or worse for random RNA sequences. We find that this is in fact the case. For example, in a random sample of 2500 sequences of length 40, Spearman's *r *= -0.58 for numbers of paired bases and log-transformed neutral network size, and Spearman's *r *= 0.54 for the contiguity statistic and log-transformed neutral network size (P < 10^-17^). However, the fractions of explained variances are less than *r*^2 ^= 0.4 and *r*^2 ^= 0.3, respectively. In sum, rapidly computed indicators of neutral network size exist, but these indicators leave the majority of neutral network size variance unexplained.

## Discussion

The method we presented to compute neutral set sizes makes direct estimation of astronomically large neutral set sizes possible for the first time, but this ability comes at a cost. With currently available computational resources, the method can accurately estimate neutral set sizes for individual RNA molecules up to length *L *= 100. If one wants to estimate the relative abundance of an RNA phenotype, this size reduces to *L *= 60 because one needs to estimate relative ranks from a sufficiently large sample of genotypes in the same sequence space, as we did in Figure 2a. Many functional RNA molecules are substantially longer than that, so computational cost is currently a limitation.

In earlier work, an RNA structure was called frequent if its associated neutral set had a size greater than that of the average neutral set [18,34]. Using our notation, such a frequent structure has a *P*-value of *P *< 0.05. The 82 biological structures from the functional RNA database [26] that we examined here are vastly more abundant than that. Their median *P*-value of 5.7 × 10^-5 ^means that fewer than 1/*P *≅ 17,500 structures are more abundant than the average biological structure. Despite their atypically large neutral sets, these networks occupy only a very small (median) fraction of 1.4 × 10^-7 ^of sequence space. These observations show that a structure may both occupy a tiny fraction of sequence space, and have a huge neutral set. The reason is simply that sequence space is unfathomably large, and has enough space for an astronomical number of structures with enormous neutral sets. Being atypically abundant and occupying a small fraction of sequence space are thus no contradictions. This would hold even more so for sequences longer than those we were able to study. When comparing neutral network sizes of biological structures to structures adopted by random *genotypes*, we found the biological structures to have somewhat larger neutral network sizes, but our sample sizes were too small to draw statistically sound conclusions.

Why are structures of biological molecules not atypically rare? Consider an evolutionary search in sequence space that is successful only if it discovers a sequence with a desirable structure, a structure that can be involved in some biological function beneficial to the organism. If both a rare and a frequent structure can satisfy these constraints, then the search will most likely find the frequent structure first. In other words, the abundance of biological structures suggests that solutions to problems that organisms face will be more readily found among abundant structures.

A high abundance of biological structures – if true generally – would have implications for the ability to find new structural variants starting from any one structure *S*. Rare structures *S *have small neutral networks. Their immediate neighborhood-defined as all sequences that differ by one nucleotide from a sequence on the network – will contain few structures different from *S*. In contrast, abundant structures have large neutral networks, in whose neighborhood many structural variants reside. If we accept that some small fraction of such variants may be novel structures beneficial to the organism – evolutionary innovations – then abundant structures may have an advantage in discovering such innovations, simply because they have access to more structural variants. A large neutral network may thus facilitate the production of useful phenotypic variation [7,33,35,36]. (Incidentally, among these structural variants, abundant structures would again be more easily found.) In addition, it has been shown that populations of RNA molecules which evolve under the influence of mutation and selection to maintain their structure, can spread more rapidly on a large neutral network. They thus gain access to a greater amount of structural variants in their immediate neighborhood [7]. All in all, structural abundance can facilitate the production of structural variation, as can other factors [27,28,37-40].

Our observations on average mutational robustness of RNA sequences also speak to the importance of neutral network size. RNA sequences with extremely high mutational robustness have few new structures in their neighborhoods [6]. One might thus think that RNA phenotypes with large neutral networks would show such extremely high robustness. However, for the 82 structures we analyzed here, mutational robustness is modest and varies by a factor of less than two (*R*_*μ *_= 0.23–0.52), whereas the corresponding neutral network sizes vary by more than fourteen orders of magnitude (1.7 × 10^11^-2.2 × 10^25^). A similar observation has been made previously in studies of random graphs that can be used as models for the RNA genotype-phenotype relationship [4]. It suggests that a modestly reduced number of neighbors with different structures in large neutral networks is much more than compensated for by the vastly increased neutral network size observed in abundant structures.

Some caveats to our findings are in order. First, while they suggest that many biological RNA sequences may have abundant structures, it is clear that there are biological RNA structures that are rare. The most prominent example is the simple stem-loop (or hairpin), which, unadorned by other structural elements, is a frequent regulatory motif, for example in translational regulation [41]. Because its many paired bases constrain its sequence severely, it is a rare structure. Second, although for some regulatory RNA molecules, only the secondary structure may be important, many RNA molecules may evolve under substantial additional constraints. Consider, for example, the hammerhead ribozyme [42], where some mutations that leave the secondary structure intact may completely abolish its biochemical activity; or the telomerase RNA, whose interaction with telomerase is critical for telomerase function [43]. For such RNA molecules, the set of mutations that do not abolish RNA function will be substantially smaller than the set of mutations that preserve the secondary structure. However, even in that case, structures with a larger neutral network to begin with may tolerate more sequence change. Third, for reasons of computational limitations, we have considered only a small sample of RNA structures. The most prominent known functional RNA structures are much longer than those we could study here, and it is an open question whether the same observations will hold for longer sequences. We hope that the method we propose here will help answer this question.

## Conclusion

We here presented a method to estimate the size of the set of genotypes that adopt a given phenotype, and to estimate the size of this set relative to other such sets.

Because the method is based on the Nested Monte Carlo approach, it can estimate neutral set sizes even where these sets are disconnected. Although we applied the method to RNA molecules, the method is general and can be applied to different systems, such as proteins or biological networks, provided that two prerequisites are met. First, for the study system it must be possible to determine a phenotype from a given genotype. The number of genotype-phenotype maps where this is possible is increasing, and includes not only molecular phenotypes (e.g., lattice proteins and simple peptides), but also phenotypes adopted by genetic networks [44-46]. Second, a notion of distance among different phenotypes must exist. This is generally not a problem because such measures can be readily defined for phenotypes as different as protein structures and gene expression patterns. The overall computational framework may also be of use in other disciplines such as computer science or engineering.

## Competing interests

The authors declare that they have no competing interests.

## Authors' contributions

AW conceived the project, TJ and OCM designed the algorithm, and TJ implemented it; all authors analyzed the data and contributed to writing the manuscript. The authors have read and approved the final manuscript.

## Supplementary Material

Additional file 1 **The effect of our sampling procedure.** The horizontal axis shows neutral network sizes, the vertical axis shows *P*-values determined in two different ways, for all 224 structures adopted by sequences of length 14. For molecules this short, all sequences can be enumerated, and neutral network sizes, as well as *P*-values can thus be determined exactly (black circles). Grey, open circles with error bars indicate estimates obtained for M = 10000 sequences through the Nested Monte Carlo method with our sampling procedure. As discussed in the main text, the biased sampling procedure preferentially identifies structures with large neutral networks. This is reflected in the higher accuracy of our estimates for large neutral network sizes (main figure), which are most relevant to the analysis of biological RNA molecules (enlargement displayed in inset). Click here for file

Additional file 2 **Cycle to cycle correlations in the Markov chain procedure.** Variation in estimated neutral network sizes during 700,000 mutation/exchange cycles for a 54 nt hammerhead structure "(((((((.(((((...))))).......(((((......)))))...)))))))" involved in the self-cleavage of peach latent mosaic viroid. Data is plotted every 2000 cycles and shows that correlations arise only on short time scales. The horizontal line indicates the mean of 8.0 × 10^22 ^over the entire window shown. The inset shows the autocorrelation function *C*(*τ*) of genotype distances at cycle *t *and *t*+*τ*: 50 cycles is enough to lose memory of the preceding genotype. Thus, the Markov chain explores efficiently all genotype space. Click here for file

Additional file 3 **The 82 biological RNA molecules used in this study.** None. Click here for file
