# Supplementary material for: Neutral network sizes of biological RNA molecules can be computed and are not atypically small
Source: BMC Bioinformatics. 2008 Oct 30;9:464. doi: 10.1186/1471-2105-9-464 (PMC2639431; doi:10.1186/1471-2105-9-464)
Supplement: Additional file 1 — The effect of our sampling procedure. The horizontal axis shows neutral network sizes, the vertical axis shows P-values determined in two different ways, for all 224 structures adopted by sequences of length 14. For molecules this short, all sequences can be enumerated, and neutral network sizes, as well as P-values can thus be determined exactly (black circles). Grey, open circles with error bars indicate estimates obtained for M = 10000 sequences through the Nested Monte Carlo method with our sampling procedure. As discussed in the main text, the biased sampling procedure preferentially identifies structures with large neutral networks. This is reflected in the higher accuracy of our estimates for large neutral network sizes (main figure), which are most relevant to the analysis of biological RNA molecules (enlargement displayed in inset). [file 1471-2105-9-464-S1.doc]

**
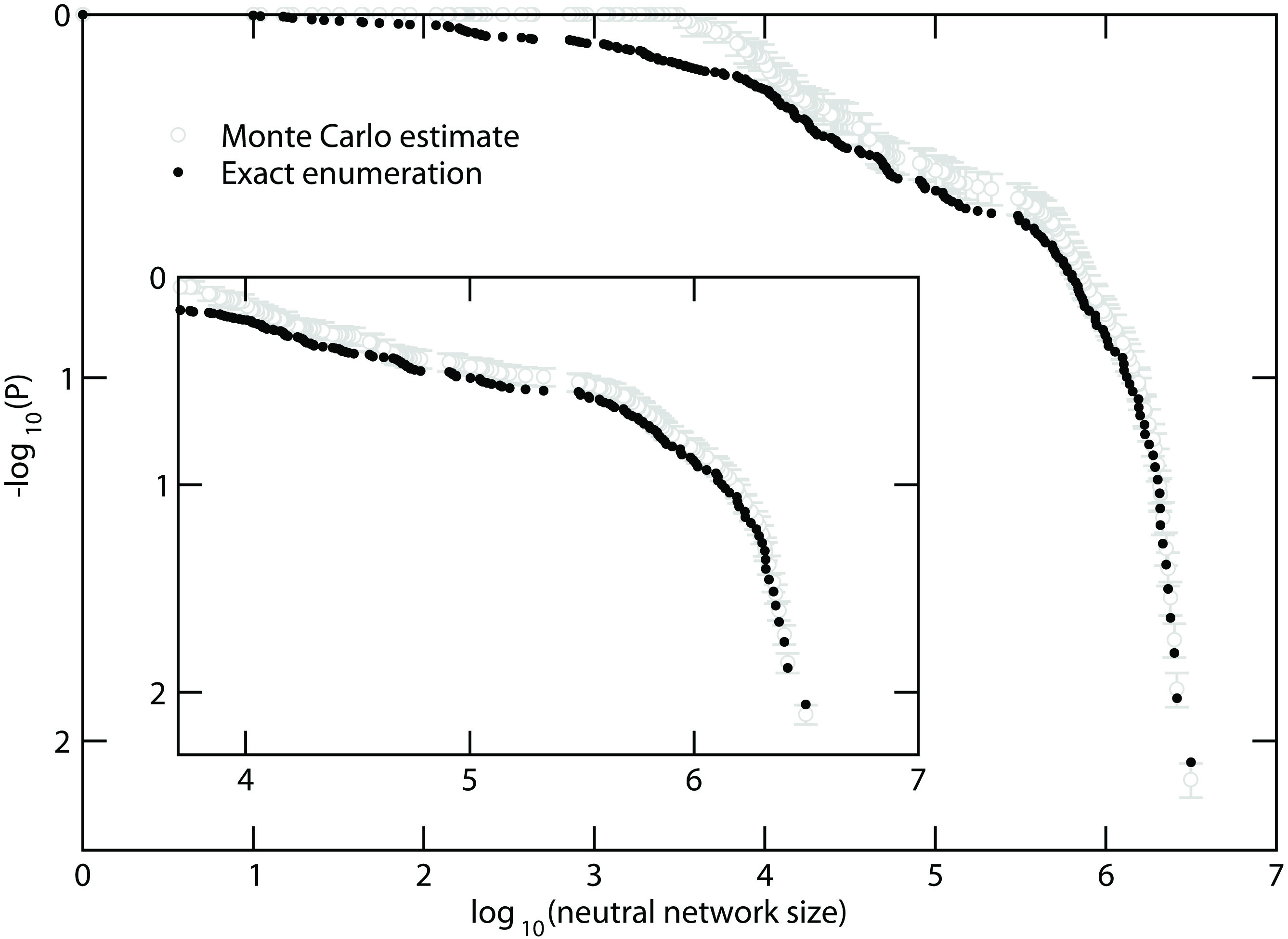
**

**Additional File 1:** **The effect of our sampling procedure.** The horizontal axis shows neutral network sizes, the vertical axis shows *P*-values determined in two different ways, for all 224 structures adopted by sequences of length 14. For molecules this short, all sequences can be enumerated, and neutral network sizes, as well as *P*-values can thus be determined exactly (black circles). Grey, open circles with error bars indicate estimates obtained for M=10000 sequences through the Nested Monte Carlo method with our sampling procedure. As discussed in the main text, the biased sampling procedure preferentially identifies structures with large neutral networks. This is reflected in the higher accuracy of our estimates for large neutral network sizes (main figure), which are most relevant to the analysis of biological RNA molecules (enlargement displayed in inset).
