# Supplementary material for: Neutral network sizes of biological RNA molecules can be computed and are not atypically small
Source: BMC Bioinformatics. 2008 Oct 30;9:464. doi: 10.1186/1471-2105-9-464 (PMC2639431; doi:10.1186/1471-2105-9-464)
Supplement: Additional file 2 — Cycle to cycle correlations in the Markov chain procedure. Variation in estimated neutral network sizes during 700,000 mutation/exchange cycles for a 54 nt hammerhead structure "(((((((.(((((...))))).......(((((......)))))...)))))))" involved in the self-cleavage of peach latent mosaic viroid. Data is plotted every 2000 cycles and shows that correlations arise only on short time scales. The horizontal line indicates the mean of 8.0 × 1022 over the entire window shown. The inset shows the autocorrelation function C(τ) of genotype distances at cycle t and t+τ: 50 cycles is enough to lose memory of the preceding genotype. Thus, the Markov chain explores efficiently all genotype space. [file 1471-2105-9-464-S2.doc]

**
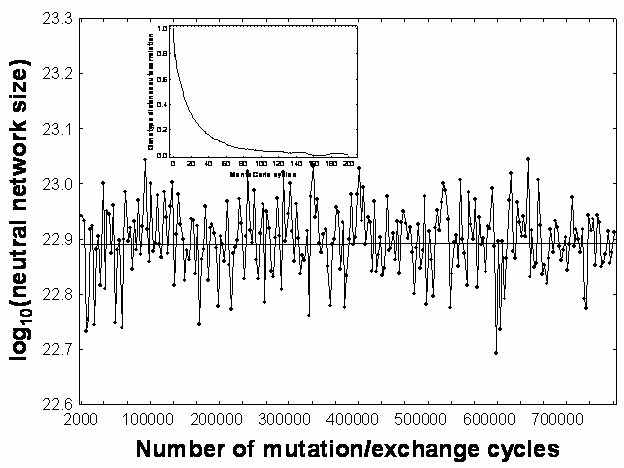
**

**Additional file 2**: **Cycle to cycle correlations in the Markov chain procedure.** Variation in estimated neutral network sizes during 700,000 mutation/exchange cycles for a 54nt hammerhead structure “(((((((.(((((...))))).......(((((......)))))...)))))))” involved in the self-cleavage of peach latent mosaic viroid. Data is plotted every 2000 cycles and shows that correlations arise only on short time scales. The horizontal line indicates the mean of 8.0×10^22^ over the entire window shown. The inset shows the autocorrelation function *C(τ)* of genotype distances at cycle *t* and *t+τ* : 50 cycles is enough to lose memory of the preceding genotype. Thus, the Markov chain explores efficiently all genotype space.
