# Supplementary material for: Neutral network sizes of biological RNA molecules can be computed and are not atypically small
Source: BMC Bioinformatics. 2008 Oct 30;9:464. doi: 10.1186/1471-2105-9-464 (PMC2639431; doi:10.1186/1471-2105-9-464)
Supplement: Additional file 3 — The 82 biological RNA molecules used in this study. None. [file 1471-2105-9-464-S3.pdf]

Tables S1: The 82 biological RNA molecules used in this study.

The biological RNAs with L=30 from the ncRNA data base.

Id	Organism	RNA type	Sequence	Secondary structure	Neutral set size	Error
AF357483	Mus musculus	snmRNA	AAGCAAUUGUUUUACUUACAGUCUGGAGAA	...(((((((.......))))).)).....	1.50×10^{13}	3.40×10^{11}
AJ430257	Archaeoglobus fulgidus	snmRNA	CAUCCGUAUAGUAUGUGUAAGAAAGGGAGG	..(((.(.(..((....))..).).)))..	5.76×10^{11}	4.75×10^{10}
AJ505722	Arabidopsis thaliana	snmRNA	CAGAAGAGCGGAUCCAAUACCAAGACUACU	.........((........)).........	1.20×10^{14}	6.64×10^{12}
Z71666	Saccharomyces cerevisiae	snoRNA	AGGCGUGUAACAUUUAUUGGUUACAACAUG	.(...((((((........)))))).)...	1.72×10^{11}	2.61×10^{10}
AB055777	Homo sapiens	Noncoding transcript	CUCUUUUACCAAGGACCCGCCAACAUGGGC	.(((((....)))))...(((......)))	8.65×10^{12}	1.36×10^{11}
D28374	Homo sapiens	Noncoding transcript	CCUUUUGGCUCUCUGACCAGCACCAUGGCG	((.....(((........))).....))..	8.66×10^{12}	3.90×10^{11}
AJ564940	Homo sapiens	Noncoding transcript	CGGAAAUCUGAAGGGAAAGUGGCAGGAAAG	......((((..(......)..))))....	3.70×10^{13}	1.17×10^{12}
X85345	Homo sapiens	Noncoding transcript	ACAUCUCAUAGGUGCCUCUGACUGAGUUGC	.((.((((((((....))))..)))).)).	1.95×10^{12}	2.46×10^{10}

Tables S1: (cont.)

The biological RNAs with L=33 from the ncRNA data base.

id	Organism	RNA type	Sequence	Secondary structure	Neutral net size	Error
AF061109	Moneuplotes crassus	Telomerase RNA	CCUCCAUUAUAUUCAAUUUGGGAAGGAUUGAAA	...........((((((((.....)))))))).	8.54×10^{15}	4.84×10^{13}
AJ430253	Archaeoglobus fulgidus	snmRNA	CAAACUUAACGAACGCUACGAGUUGAAAUCAGA	.....(((((...........)))))).......	9.02×10^{15}	1.71×10^{14}
S80603	Trypanosoma cruzi	gRNA	GAUAGAGAACGGAUGAGAAGGAUGUUUGUGAGA	........((((((.........))))))....	1.03×10^{16}	1.45×10^{14}
AB017710	Homo sapiens	snoRNA	UAAUCAAUGAUGAAACCUAUCCCGAAGCUGAUA	........((((.....)))).......... ..	2.92×10^{16}	5.28×10^{14}
AB055770	Homo sapiens	Noncoding transcript	CUUUCUGGUCUCGGCCGCAGAAGCGAGAUGACG	.......(((((...(((....))).)).))).	5.34×10^{13}	1.66×10^{12}
AB056456	Homo sapiens	Noncoding transcript	CUCCUCUCUCCGGUCCGUGCCUCCAAGAUGACA	.((.(((....(((....)))....))).))..	1.27×10^{14}	3.47×10^{12}
D28386	Homo sapiens	Noncoding transcript	CUUCCUUUCCAACUUAGACGCUGCAGAAUGGCU	...................(((.......))).	1.87×10^{16}	5.78×10^{14}
S43366	Homo sapiens	Noncoding transcript	GCCUUGAAGGGAGGUUUGUAGAAAACUGUAAGC	.(((....)))..((((((((....))))))))	1.43×10^{14}	1.28×10^{12}
AJ309206	Homo sapiens	Noncoding transcript	CCCGAAACUCUGGAUUCCGGGGCCCUUCACAGC	...(((.((((((...))))))...))).....	1.45×10^{14}	3.03×10^{12}

Tables S1: (cont.)

The biological RNAs with L=35 from the ncRNA data base.

id	Organism	RNA type	Sequence	Secondary structure	Neutral net size	Error
AF380696	Leishmania tarentolae	gRNA	AAACAAACAGUGCACGGGGUCGGAUGUUAAACACA	.....((((...(.((....))).))))).......	1.11×10^{14}	7.97×10^{12}
AJ430256	Archaeoglobus fulgidus	snmRNA	GCGAGAGUUUAGGCGGCUUGUAAGAAAGGGAGGCU	(((((.((....))..)))))..............	2.28×10^{16}	4.36×10^{14}
AJ544037	Drosophila melanogaster	snmRNA	GCCCGAAGAAGCCGAAAGCUUGGAUGGCUGGGAUG	.((((......((((....)))).....))))....	5.37×10^{15}	1.13×10^{14}
AJ544038	Drosophila melanogaster	snmRNA	CAUUUGGAGAACGAAAGAAGACCAACAAGUGCGCG	((((((.....(....)........))))))....	2.36×10^{14}	2.72×10^{13}
L11652	Trypanosoma brucei	gRNA	CCAACAAACAGAAUAACUAGUGCACGGUGAUGAUG	........((..((.(((.......))).))..))	1.95×10^{15}	7.98×10^{13}
M11772	Homo sapiens	snRNA	ACCCAAUUUUUUGAGGCCUUGCUUUGGCAAGGCUA	.............((((((((....)))))))).	1.28×10^{17}	6.97×10^{14}
AB055765	Homo sapiens	Noncoding transcript	CUUCUUCCUUCUCGCCUAACGCUGCCAACAUGGUG	.......................(((.....))).	3.51×10^{17}	6.91×10^{15}
D28349	Homo sapiens	Noncoding transcript	UUCCUUUUUUCUUUUUUCCGGCGUUCAAGAUGUCG	.................(((((((...)))))))	9.73×10^{16}	8.58×10^{14}

Tables S1: (cont.)

The biological RNAs with L=40 from the ncRNA data base.

Id	Organism	RNA type	Sequence	Secondary structure	Neutral set size	Error
AF380736	Leishmania tarentolae	gRNA	AACCACACAGAGAACAAUUUACACGUAGAAUGCAACAUUG	…...........((.((((….)))).))…......	1.82×10^{19}	5.08×10^{17}
AJ430248	Archaeoglobus fulgidus	snmRNA	GCCGAAUCGAUCAGAAGAGAACUGACAAGUUGAAAUCAGA	…...((((((((…....))))….))))….....	1.12×10^{18}	2.54×10^{16}
AJ430294	Archaeoglobus fulgidus	snmRNA	CAACGCUGCAACAAGAGUGGUUUUACCGUGAUCAACGUGA	…((((…......))))…...((((…..))).).	4.45×10^{15}	7.52×10^{14}
L25589	Trypanosoma brucei	gRNA	GACACAAUGAAGAAAUGCACUCUAAAAUGCACAGUGAUUA	…............((((…......))))…......	1.17×10^{20}	3.74×10^{18}
L25590	Trypanosoma brucei	gRNA	UAAAAGACAAUGUAGAUUUCUGAGUAAUAGGGAGGAUAAC	…..........((..(((((.(….).)))))..))..	9.13×10^{17}	3.30×10^{16}
D28454	Homo sapiens	Noncoding transcript	CUUUCCGGCGGUGACGACCUACGCACACGAGAACAUGCCU	…...((((….)).))…(((…........)))..	9.19×10^{15}	7.08×10^{14}
X99684	Homo sapiens	Noncoding transcript	CAGGCCGCUGUCCUCCUGCUCCUACCCACCUAGUGUCAAG	.(((..((…......)).)))…...............	7.28×10^{17}	6.05×10^{16}

Tables S1: (cont.)

The biological RNAs with L=43 from the ncRNA data base.

id	Organism	RNA type	Sequence	Secondary structure	Neutral set size	Error
AF036740	Schistosoma mansoni	Ribozyme	AUCCAGCUCACGAGUCCCAAAUAGGACGAAACGCGUCCUCCAU	.....(((....))).......((((((.....)))))))....	5.84×10^{19}	1.75×10^{18}
X15621	Schistosoma mansoni	Ribozyme	AUCCAGACGCGCGUUCGUCUUAUUUGGGACUCGUCAGCUGGAU	((((((..(((.((((..........)))).)))...))))))	2.19×10^{18}	5.06×10^{16}
AF380675	Leishmania tarentolae	gRNA	AAUACGCAUAAUUACUAGAUCGUGAAUAGAAGAGAUAGAUAUU	..............(((..((..........))..))).....	1.34×10^{20}	6.96×10^{18}
AF380679	Leishmania tarentolae	gRNA	ACUCCUGGCAGAUUAAUAGAACUAUCACUCUCGGUUGUAGACA	.....................((((.((.....)).))))...	9.38×10^{20}	2.92×10^{19}
AF380717	Leishmania tarentolae	gRNA	UUUAUGCUUGACAGACUAACAAUACUACUUUUAACUAGAGACG	...........................((((.....))))...	6.29×10^{21}	1.75×10^{20}
AF380724	Leishmania tarentolae	gRNA	ACUCCUGGCAAGUCAAUAGAAUUAUCGUCUCCGAUUAUAGAUA	.((..(((....)))..))....((((....)))))........	1.19×10^{19}	4.64×10^{17}
AJ430285	Archaeoglobus fulgidus	snmRNA	GCGGGAUCUGCAUUGUUUCACCCAGAGGAGGCUUACGCCUCCU	..(((....((...))....)))..(((((((....)))))))	9.34×10^{16}	5.85×10^{15}
AJ544018	Drosophila melanogaster	snoRNA	GCUUCACCCAGAGCAGGGCAGUGAAAAAUGGUCAAGCACAAAU	((((.(((....((......)).......))).))))......	2.09×10^{18}	1.56×10^{17}
AJ544036	Drosophila melanogaster	snmRNA	GCGGCGGCGGCCGUUCAACAUGUGUUGUUUAACUUUGUGGCCA	..(((.((((..(((.((((.....)))).))).)))).))).	1.16×10^{18}	2.60×10^{16}
S80581	Trypanosoma cruzi	gRNA	AAACAAAACAUAAAGAUAGGUGAAGUGAAUAUUGAGAGAUUUG	...((...(((........)))...))................	9.98×10^{19}	7.23×10^{18}
U12393	Trypanosoma brucei	gRNA	AUAUAAGUACAAACAGACAGUGAGAAGAUAACGAGACUGAGUA	.................((((..............))))....	1.24×10^{21}	5.01×10^{19}
D28352	Homo sapiens	Nc. transcript	CUUUUUCCGGUGUGGAGUCUGGAGACGACGUGCAGAAAUGGCA	((.((((..(..((..(((....)))..))..).)))).))..	8.98×10^{16}	5.70×10^{15}

Tables S1: (cont.)

The biological RNAs with L=50 from the ncRNA data base.

id	Organism	RNA type	Sequence	Secondary structure	Neutral net size	Error
AJ248286	Pyrococcus abyssi GE5	snRNA	UAUGAUGAGAGAGCUGUUAAAUGAUUGAUGAUUAGAGGAUGGCUGGCUGA	.......((..(((((((...(((((...)))))...)))))))..))..	6.37×10^{21}	1.67×10^{20}
AJ248285	Pyrococcus abyssi GE5	snRNA	AAUGAUGAAAUCGGUCUUGUCCGAGGAGUGAUGAUAGUGGUCACCGCUGA	..........((((......))))..((((.((((....)))).))))..	6.57×10^{22}	1.40×10^{21}
AJ248283	Pyrococcus abyssi GE5	snRNA	AAUGAUGAGACCGGCACUAACUGAGUUGUGAUGAGCACUCGGUUGGCUGA	...........((((.(.((((((((.((.....))))))))))))))).	1.16×10^{21}	3.33×10^{19}
AJ248287	Pyrococcus abyssi GE5	snRNA	GAUGAUGAGGCCUACCUCGCUGAGCUGUGAUGAGUGGGUGAGCUGGCUGA	........((((...((((((..(((......))).))))))..))))..	1.82×10^{22}	5.39×10^{20}
AF254841	Pyrococcus abyssi GE5	snoRNA	GAUGAUGAGCGCCAUCGAUGCUGAGGAGUGAUGACCGGAUUCCUGGCUGA	..(((((.....)))))..(((.((((((.........)))))))))...	9.17×10^{21}	1.94×10^{20}
AF254842	Pyrococcus abyssi GE5	snoRNA	GAUGAUGAGCGUUUACCGGUCUGAGCUGUGAUGACAUCGGCACUGUCUGA	..(((((..((((...((((....)))).)))).)))))...........	2.93×10^{22}	7.66×10^{20}
AJ248283	Pyrococcus abyssi GE5	snRNA	AAUGAUGAGAUGGCGGAUUGCUGAGCUGUGAUGAAGACCUUAGGGUCUGA	.........((.((((.........)))).))..(((((....)))))..	3.07×10^{22}	1.15×10^{21}
AJ248283	Pyrococcus abyssi GE5	snRNA	GAUGAGGAAGCCGAGGACACCUGAAUUGUGAUGAAUCUUCGCUUCGCCGA	......(((((.((((((((.......))).....)))))))))).....	4.49×10^{21}	1.26×10^{20}
AJ248283	Pyrococcus abyssi GE5	snRNA	UCAGGUGAUGCGGACUUCAUCACCACUCAGCCGGUUGAUCCAUCAUCAUC	...(((((((.(((..(((..(((........)))))))))..)))))))	6.62×10^{19}	2.51×10^{18}
AJ248283	Pyrococcus abyssi GE5	snRNA	UCAGCCAGUAUGCCCUUCAUCACCCUUCAGCAACAAGCGAGUUCAUCAUC	...((..((.(((...............)))))..)).............	1.50×10^{22}	1.34×10^{21}
AJ248284	Pyrococcus abyssi GE5	snoRNA	UCAGCUAAUACCGACAUCAUCACCUUUCAGGCUGGGCAAGGCUCGUCAUC	.............................(((.((((...)))))))...	5.55×10^{23}	1.62×10^{22}
AJ248284	Pyrococcus abyssi GE5	snRNA	UCAGCCCAUGCGAGUUUCCUCACCUUUCGGCCGAAAUCUCCUUCAUCAUC	((.(((...(.(((....))).).....))).))................	2.63×10^{21}	3.03×10^{20}
AJ248284	Pyrococcus abyssi GE5	snRNA	GAUGAUUGGAAACGGAGUGAGCUGAGGUGUGAUGACCGACUUCCCGCUGA	.....(((....)))....(((.(((((.((.....)))))))..)))..	6.96×10^{20}	3.36×10^{19}
AJ248284	Pyrococcus abyssi GE5	snRNA	UCAGUUGCGAGGGUACUCAUCACUUUUCAGCUAGCGAAAUUUUCUUCAUC	..(((((.((((((.......)))))))))))...(((......)))...	5.82×10^{21}	1.24×10^{20}
AJ248285	Pyrococcus abyssi GE5	snRNA	GAUGAGGAGUUCAGCCUAGGCUGAGGCGUGAUGAGGGAGUGACGGACUGA	.........((((((....)))))).(((.((......)).)))......	3.72×10^{22}	1.43×10^{21}
AJ248286	Pyrococcus abyssi GE5	snRNA	UCAGGGGGUGUUCACCUCAUCAUAGGUCAGGUGGCUUGCUACUCAUCAUC	...(((((((.((((((.((.....)).))))))..)))).)))......	2.28×10^{21}	7.06×10^{19}
AJ248287	Pyrococcus abyssi GE5	snRNA	UCAGGAAGCCGUCCACUCCUCACCGUUCAGGUGCGGAAGGCUUCAUCAUU	....((((((......(((.((((.....)))).))).))))))......	1.41×10^{22}	4.01×10^{20}
AJ248287	Pyrococcus abyssi GE5	snRNA	GAUGAGGAGAGGGUAGGUAGCUGAGUUAUGAUGAACUAAUCGGCCCCCGA	..........(((..(((.....((((......)))).....))))))..	1.11×10^{22}	6.90×10^{20}
AJ248287	Pyrococcus abyssi GE5	snRNA	UCAGAACCACGAGACAUCAUCACACUUCAGCCAAGGGUGGGAUCAUCCUC	.................................((((((....)))))).	2.24×10^{25}	5.21×10^{23}
AF357428	Mus musculus	snoRNA	GAUCUGAUGGGGNCUGAGUGUUUCUCGNUGACAUGAACAGAUAUCUGAGG	..((.((((....(((.(((((.......)))))...))).)))).))..	5.16×10^{20}	2.11×10^{19}
AF357500	Mus musculus	snmRNA	CAGGAAGAGAUGAAAAUUCUAAGAGAGGUCCUCAGAAAAUCAAAAGAAGA	..........(((...((((..(((.....)))))))..)))........	1.62×10^{22}	6.55×10^{20}

Tables S1: (cont.)

The biological RNAs with L=50 from the ncRNA data base (cont.)

AF380713	Leishmania tarentolae	gRNA	UAAAACAUCAAACAAAAAGUUGAGUCACAGAUAAAUGAGAGACACGUUAG	...(((....(((.....)))..(((.((......))...)))..)))..	5.55×10^{19}	5.12×10^{18}
AF380749	Leishmania tarentolae	gRNA	AUACAACGGCGAUAACUAAUAUUAGACAUAGCGGAAAAUAGAUAAUUAUA	......(.((.....(((....)))......)).)................	3.65×10^{21}	1.11×10^{21}
M10126	Leishmania tarentolae	gRNA	ACAUAUGACUUGAAGUUAAAAGAUAAUAUAAAUUUUUAAAUAAUAACUUA	...........(((((((((((.......))))))......)))))).	1.66×10^{23}	2.85×10^{21}
X60509	Leishmania tarentolae	gRNA	GUUAAACAUUAACUCGGUAUAAAUAUAGGGAACAUUGUAGAGUCGUAUAU	((((.....)))).........(((((.((..(......)..)).)))))	4.87×10^{21}	2.84×10^{20}
AJ430259	Archaeoglobus fulgidus	snmRNA	UGGUGACUUGGCAACCAUCUUGUAAGAAAGGGAGGCUCCUGAAAAUGGAG	.(....).......((.((((......))))..))((((.......))))	2.01×10^{17}	5.91×10^{16}
L16537	Trypanosoma brucei	gRNA	UAUAAAUACCACAUGAAUCUUAUAUGUACGAUGGAAGAUGAGAAUUAUGC	..........(((((.((((((...(......)...)))))).))))).	1.81×10^{22}	9.16×10^{20}
L16541	Trypanosoma brucei	gRNA	AUAUAAACAAACAACUAAUAAAAUGUAAAUCUGAUGUGUGAUAUACUAUG	.......................(((.(((........))).))).....	3.02×10^{24}	1.23×10^{23}
L16543	Trypanosoma brucei	gRNA	AUAUAACCAAAAACACUGCGAUUUGAUGUAAUAAGUGACUGUGUAAAUUU	...........((((.(..(((((......)))))..).))))......	1.52×10^{23}	5.67×10^{21}
M17439	Tobacco ringspot virus	Ribozyme	UACCAGGUAAUAUACCACAACGUGUGUUUCUCUGGUUGACUUCUCUGUUU	.((((((.(((((((......))))))).).))))).............	3.98×10^{22}	1.11×10^{21}
S80611	Trypanosoma cruzi	gRNA	GGUGUAUAUGGUGACAAACUAUGAAAGGUAGAGUUGUGGAGUUGAUAGAA	..(.(((....(.((((.((((.....))))..)))).).....))).).	1.51×10^{19}	2.09×10^{18}
U30216	Crithidia fasciculata	gRNA	UCUCUCCCUAUAAUAAAAACAUUAUAGGGAGAGAGUAAAGGUGCCCUAUA	((((((((((((((......)))))))))))))....(((...)))...	7.05×10^{20}	1.90×10^{19}
U30217	Crithidia fasciculata	gRNA	GAGCCACGGUUCUAGAUCGAACCAUGUGUAUAGUACAGGCAUAACUAUUU	..(((..((((((......))))))..(((.....))).))).........	9.00×10^{21}	3.44×10^{20}
AB055774	Homo sapiens	Noncoding transcript	CUCUUCCUAAGCCGGCGCUCGGCAAGUUCUCCCAGGAGAAAGCCAUGUUC	..........((((.....))))...(((((....)))))..........	3.03×10^{23}	8.57×10^{21}
AF082503	Homo sapiens	Noncoding transcript	CGACCAGCCGAAUCGCUCCUGCAGCAGAGGCCGAUACAAAGCAGGGGAAA	..............(((((((..................)))))))...	4.56×10^{24}	1.57×10^{23}
AF279138	Homo sapiens	Noncoding transcript	CCCGCCAGAGACAGCUCAGGUGCAAGGAGAUGAGACUAUACAUCAGGCAG	...(((.(((....)))...........((((........)))).)))..	6.60×10^{21}	2.97×10^{20}
S60300	Homo sapiens	Noncoding transcript	GGGUGUUUAUUCCUCAUGGACUAAUUAGGAACAGGACUGAACGUCUUGCU	((..(((((.((((......((....))....)))).)))))..)).....	1.50×10^{21}	7.44×10^{19}
X58756	Homo sapiens	Noncoding transcript	UGGAAACAAGCUGGUCUUUGGCGCAGGAACCAUUCUGAGAGUCAAGUCCU	((....))....((.(((.(((.(((((....)))))...)))))).)).	6.40×10^{19}	4.08×10^{18}
